# circ-TFRC downregulation suppresses ovarian cancer progression via miR-615-3p/IGF2 axis regulation

**DOI:** 10.1186/s12935-024-03287-4

**Published:** 2024-04-27

**Authors:** Zhongxin Yan, Changling Duan, Xi Li, Hao Wang, Shanji Li, Xuexin Zhou, Yi Miao

**Affiliations:** Department of Obstetrics and Gynecology, Shanghai General Hospital, Shanghai Jiaotong University School of Medicine, Shanghai, 200080 China

**Keywords:** circ-TFRC, IGF2, miR-615-3p, Ovarian cancer

## Abstract

**Background:**

Ovarian cancer (OC) is a malignancy among female globally. Circular RNAs (circRNAs) are a family of circular endogenous RNAs generated from selective splicing, which take part in many traits. Former investigation suggested that circ-TFRC was abnormally expressed in breast cancer (BC). Further, the role of circ-TFRC to the progress of OC remains unclear. So, the aim of this study was to reveal the regulatory mechanism of circ-TFRC.

**Methods:**

Our team made the luciferase reporter assay to validate circ-TFRC downstream target. Transwell migration assay, 5-ethynyl-20-deoxyuridine, and cell counting kit-8 were applied to investigate both proliferation and migration. In vivo tumorigenesis and metastasis assays were performed to investigate the circ-TFRC role in OC.

**Results:**

The outputs elucidated that circ-TFRC expression incremented in OC cells and tissues. circ-TFRC downregulation inhibited OC cell proliferation as well as migration in in vivo and in vitro experiments. The luciferase results validated that miR-615-3p and IGF2 were circ-TFRC downstream targets. IGF2 overexpression or miR-615-3p inhibition reversed OC cell migration after circ-TFRC silencing. Also, IGF2 overexpression reversed OC cell migration and proliferation post miR-615-3p upregulation.

**Conclusion:**

Results demonstrate that circ-TFRC downregulation inhibits OC progression and metastasis via IGF2 expression regulation and miR-615-3psponging.

## Background

Ovarian cancer (OC) is a malignancy involving female reproductive system, resulting in high mortality compared with other gynecological malignancies [1]. Despite improvements of surgery treatment and chemotherapy, five-year survival of OC patients is merely about 30% because of frequent relapses [2, 3]. Therefore, the primary goal of OC therapy is to recognize potential prognostic markers that could be applied to distinguish patients with high relapse risks and biomarkers as potential therapy targets. Former investigations found that circular RNAs (circRNAs) have a significant role in cancer development, which have therapy and prognostic values [4, 5]. Nevertheless, the mechanisms of molecular circRNA regarding OCs are unclear.

circRNAs are a class of endogenous RNAs identified by continuous rings that are covalently closed. They possess indispensable characteristics to develop different malignancies such like gastric cancer, hepatocellular carcinoma, cervical cancer, breast cancer (BC), and OC [5–10]. A previous study confirmed that hsa_circ_0068631 (circ-TFRC) expressed highly in BC cells and tissues. Upregulation of circ-TFRC may facilitate BC progression through EIF4A3 binding to maintain c-Myc mRNA stability [11]. Previous studies have confirmed the transferrin receptor (TFRC), also known as CD71, is an essential membrane protein regulating intracellular iron transport [12, 13]. Although several reports have documented abnormal TFRC overexpression in some human tumors, such as anaplastic thyroid carcinoma and astrocytic brain tumor [14, 15]. TFRC expression was significantly increased in human OC tissues, especially in metastases, and this overexpression of TFRC was directly associated with poor prognosis [16]. But if circ-TFRC play a role in the progression of OC remains unknown. Therefore, present investigation was to unravel regulatory mechanisms of circ-TFRC regarding patients with OC.

We analyzed human OC tissue. Data revealed that circ-TFRC was upregulated in OC, and circ-TFRC silencing inhibited cell migrations in the experiments. Furthermore, current study revealed that circ-TFRC enhanced the malignant progression of OC by promoting IGF2 expression and miR-615-3p sponging. This study provides insights for the clinical applications of new therapeutic strategies in treating OC.

## Methods

### Ethics statement

Our team employed BALB/c nude female mice aged 4 weeks with 15 ∼ 20 g weight (SLARC, Shanghai, China). Animal Research Committee in the Shanghai First People’s Hospital, affiliated with Shanghai Jiao Tong University (SJTU), approved animal protocols.

### Tissue chips and FISH

We gained OC tissue chips (*n* = 10) from the Shanghai First People’s Hospital affiliated with SJTU. Geneseed Biotech (CA, USA) provided particular probes for circ-TFRC (Dig-5′-GATCGTCTTTTTCTTGATTGGTTCTTCTGTG-3′-Dig). For FISH analyses, the technician captured signals and counterstained nuclei applying DAPI (Yeasen Biotechnology, Shanghai, China) for 15 min. Technician obtained photos utilizing Zeiss LSM 700 confocal microscope (Carl Zeiss GmbH; Oberkochen, Germany).

### Cell culture

Our team obtained 4 human OC cells (OVCAR-3, SKOV3, A2780, and ES-2) and 1 normal epithelial cell line (IOSE80) from Chinese Academy of Sciences Cell Bank (Shanghai, China). The cells were cultured in DMEM (Gibco, MD, USA) using 10% fetal bovine serum (FBS) (Gibco) and 50 µg/mL streptomycin at 37 °C in a humidified atmosphere with 5% CO_2_.

### Bioinformatics analysis

We used the Encyclopedia of RNA Interactomes database to evaluate interactions among miRNA, mRNA, and circRNA.

### RNA overexpression or interference

miR-615-3p inhibitor, miR-615-3p mimic, siRNA against circ-TFRC (si-circ-TFRC, 5′-CTGTGTGGCAGTTCAGAATGATG-3′) were purchased from GenePharma (Shanghai, China). and IGF2 overexpression vector (constructed the IGF2 cDNA sequence into the cDNA3.1 vector) were used for transfections.

### Cell migration assay

This study used 24-well Transwell chambers (BD Biosciences, NJ, USA) for cell migration analyses. We plated A2780 and SKOV3 cells (1 × 10^5^) into upper chamber and 500 µL of DMEM with 20% FBS into lower chamber. We cultured cells for 1 day at 37 °C before being fixed to the bottom chamber cells for 0.5 h with 4% paraformaldehyde. Subsequently, technician stained cells with 0.1% crystal violet (Shanghai Yisheng Biotechnology, Shanghai, China). We obtained photographs using Zeiss Axio Observer D1 microscope.

### 5-ethynyl-20-deoxyuridine analysis

The technician employed 5-ethynyl-20-deoxyuridine (EdU) assay kits (Thermo Fisher Scientific) to analyze cell activities. We maintained SKOV3 and A2780 cells (1 × 10^5^) in six-well plates for 2 days in prior putting EdU to wells for 2 h. We fixed the cells using 4% formaldehyde.

### Cell counting kit-8 assay

The technician incubated A2780 and SKOV3 cells in 10% cell counting kit-8 (CCK8) diluted in a normal culture medium at 37 °C. The proliferation rates were recorded after 0, 1, 2, and 3 days of transfection. Absorbance was recorded by employing microplate reader.

### RT-qPCR

RNA from tumor tissues and cells were isolated using TRIzol kit (Thermo Fisher Scientific). Total RNAs (500 ng) were reverse transcribed to cDNA with a PrimeScript Reverse Transcriptase Kit (Takara, Dalian, China). Further, miR-615-3p, circ-TFRC and IGF2 were detected by quantitative reverse transcription PCR (qRT-PCR) using a Bulge-Loop TM miRNA qRT-PCR Starter Kit (Applied RiboBio Biotechnology, Guangzhou, Guangdong, China) and a SYBR Green PCR Kit (Takara), respectively. Our team utilized 2^−ΔΔCT^ approach to capture fold changes in relative expression. Primers that employed in current study were:

- circ-TFRC:5′-GATCAAGCTAGATCAGC-3′ (forward) and 5′-GCTGAACCGGGTATATGAC-3′ (reverse); miR-615-3p:5′-CCGCGCATCTGGAGGTAAGAAG-3′ (forward) and 5′-AGTGCAGGGTCCGAGGTATT-3′ (reverse);
- *IGF*2:5′-GTGGCATCGTTGAGGAGTG-3′ (forward) and 5′-CACGTCCCTCTCGGACTTG-3′ (reverse);
- *U*6:5′-CTCGCTTCGGCAGCACA-3′ (forward) and 5′-AACGCTTCACGAATTTGCGT-3′ (reverse);
- *GAPDH*:5′-AATGGGCAGCCGTTAGGAAA-3′ (forward) and reverse: 5′-TGAAGGGGTCATTGATGGCA-3′ (reverse).

### Dual-luciferase reporter assay

The technician cloned putative miR-615-3p-binding sites of the wild type (wt) or mutant (mut) 3’ UTR *IGF2* and circ-TFRC into psi-CHECK (Promega, WI, USA) vector downstream. Firefly luciferase 3’ UTR or circ-TFRC was primary luciferase signal. We named these clones IGF2-Wt/circ-TFRC-Wtand IGF2-Mut/circ-TFRC-Mut. We employed the psi-CHECK vector with the Renilla luciferase signal for normalization to compensate for the variances between harvested efficiencies and transfections. This study involved HEK293 cell transfections applying Lipofectamine 2000. We detected firefly luciferase and Renilla actions 1 day after transfection.

### Tumor xenograft formation

Wt or 2 × 10^6^ sh-circ-TFRC SKOV3 cells were tail vein injection into the mouse’s right flank to calculate tumor sizes each five days for 1 month utilizing Vernier caliper. Tumor volume was computed utilizing formula: length × width^2^ × 0.5. The relative expression of Ki67 was measured employing the IH method. Each group have 5 mice.

We stably transfected luminescence-labeled SKOV3 cells with negative control (NC) for metastasis analyses. Technician suspended sh-circ-TFRC, which we injected into each nude mouse tail vein. After four weeks, our team validated lung metastasis by applying bioluminescence imaging system. The technician counted lung tissue metastatic foci after hematoxylin and eosin staining. Each group have 5 mice.

### Statistics analysis

We represented data by mean ± standard deviation (SD). GraphPad Prism (GraphPad, CA, USA) was employed to gain variances among groups. *P* value ≤ 0.05 indicated a statistics significance.

## Results

### circ-TFRC expression increased in OC tissues and cells

**Fig. 1:**
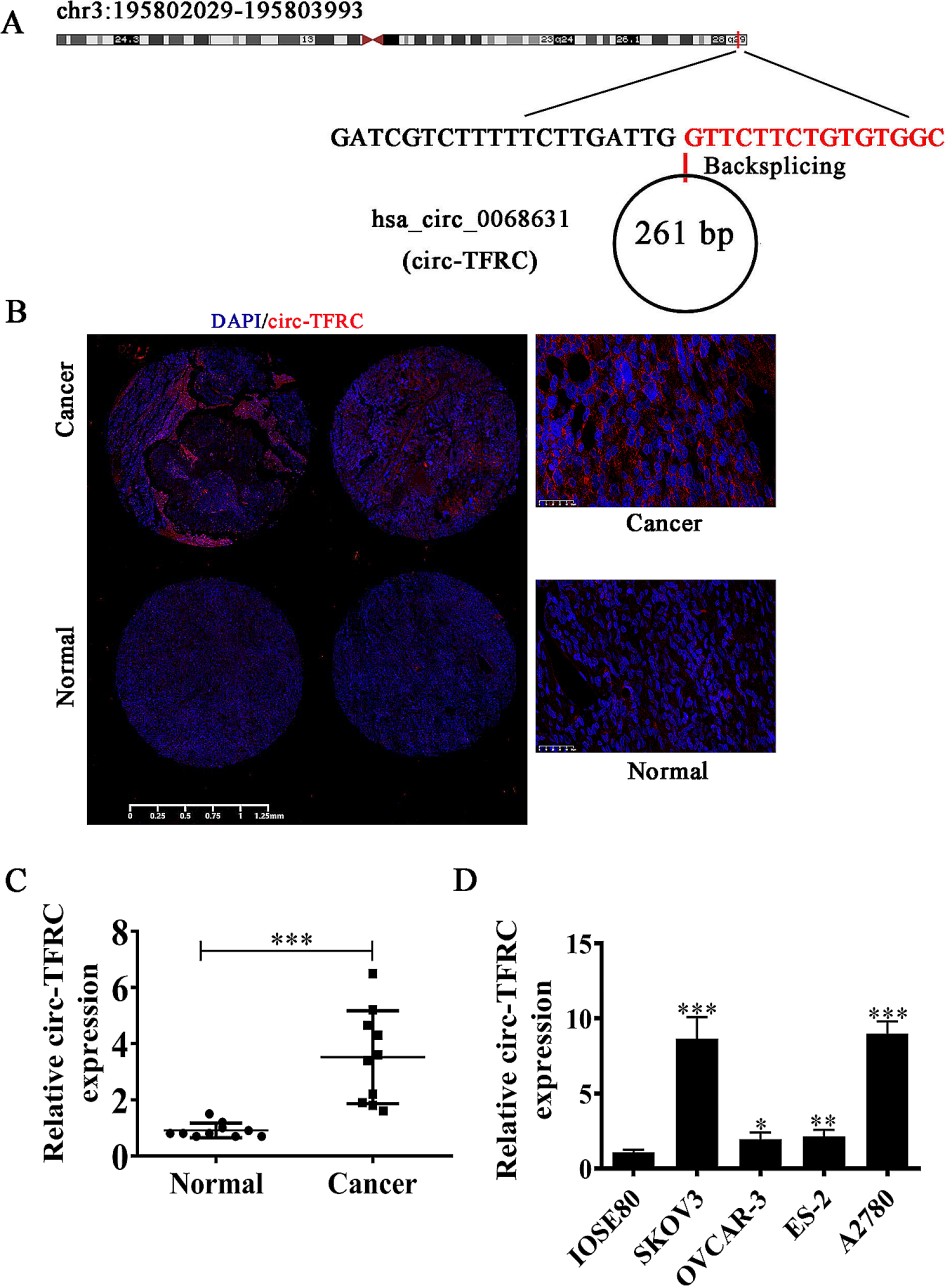
Expression of circ-TFRC increased in both OC tissues and cell lines. (**A**) Chromosomal location of circ-TFRC. (**B** and **C**) FISH detection shows the expression and subcellular localization of circ-TFRC in OC tissues. The data are expressed as the mean ± SD. ^***^P < 0.001 versus NC. (**D**) RT-qPCR detection shows the expression of hsa_circ_0013561 in normal cells IOSE80 and OC cell lines SKOV3, OVCAR-3, ES-2, and A2780. The data are expressed as the mean ± SD. ^*^P < 0.05, ^**^P < 0.01, ^***^P < 0.001 versus IOSE80. *n* = 3

This study revealed that hsa_circ_0068631 was produced by cyclizing exons from *TFRC* gene located on chr3:195802029–195,803,993. The TFRC was 1964 bp, and spliced mature circRNA was 261 bp (Fig. 1A). hsa_circ_0068631 was also called circ-TFRC. FISH data showed that circ-TFRC was localized in cytoplasm (Fig. 1B), and circ-TFRC expression increased in primary OC comparing to normal tissues (Fig. 1C). RT-qPCR outputs unraveled that circ-TFRC expression increased in OC cells OVCAR-3, SKOV3, A2780, and ES-2 comparing to IOSE80. Further, A2780 and SKOV3 cells showed much higher circ-TFRC expression (Fig. 1D). Therefore, both A2780 and SKOV3 cells were selected for following study.

### circ-TFRC downregulation inhibited OC cell proliferation and tumor growth

**Fig. 2:**
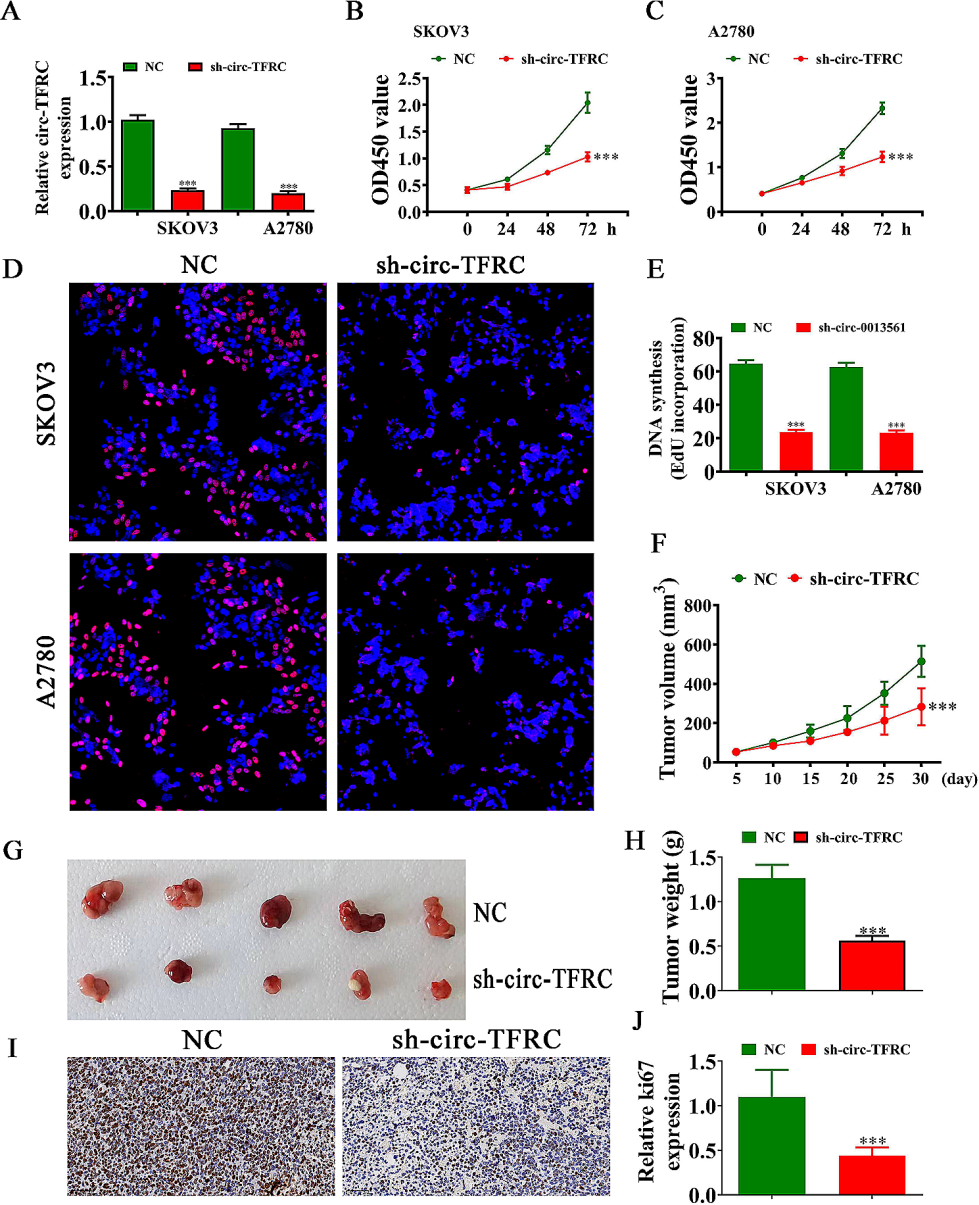
Downregulation of circ-TFRC inhibited OC cell proliferation and tumor growth. (**A**) RT-qPCR detection shows the expression of circ-TFRC in both SKOV3 and A2780 cells after transfection with NC and circ-TFRC silence vector (sh-circ-TFRC). The data are expressed as the mean ± SD. ^***^P < 0.001 versus NC. *n* = 3. (**B** and **C**) CCK8 detection showed the proliferation of both SKOV3 and A2780 cells. The data are expressed as the mean ± SD. ^***^P < 0.001 versus NC. *n* = 3. (**D** and **E**) EdU detection showed the proliferation of both SKOV3 and A2780 cells. The data are expressed as the mean ± SD. ^***^P < 0.001 versus NC. *n* = 3. (**F**) Summary of tumor volumes. The data are expressed as the mean ± SD. ^***^P < 0.001 versus NC. (**G**) Representative photographs of SKOV3 tumor formation in xenografts of nude mice. *n* = 5. (**H**) Summary of tumor weight in mice. The data are expressed as the mean ± SD. ^***^P < 0.001 versus sh-NC. (**I** and **J**) IH showed the percentage of Ki-67-positive cells. The relative number of Ki-67-positive cells was calculated. The data are expressed as the mean ± SD. ^***^P < 0.001 versus NC. *n* = 3

SKOV3 and A2780 cell transfections were used to construct siRNA against circ-TFRC (sh-circ-TFRC). RT-qPCR outputs demonstrated that circ-TFRC expression decremented significantly in the cells after sh-circ-TFRC transfection compared with NC (Fig. 2A). CCK8 (Fig. 2B, C) and EdU assays (Fig. 2D and E) showed that circ-TFRC silencing suppressed SKOV3 and A2780 cell proliferation. Xenograft mouse assay in vivo showcased tumor volume and weight (Fig. 2F-H) in nude mice injected with SKOV3 cells; the weight and volumes were reduced after sh-circ-TFRC transfections. IH detection showed that circ-TFRC downregulation suppressed Ki67 expression in tumor tissues (Fig. 2I,J), suggesting that circ-TFRC downregulation inhibited OC cell proliferation.

### circ-TFRC downregulation inhibited OC lung metastasis

**Fig. 3:**
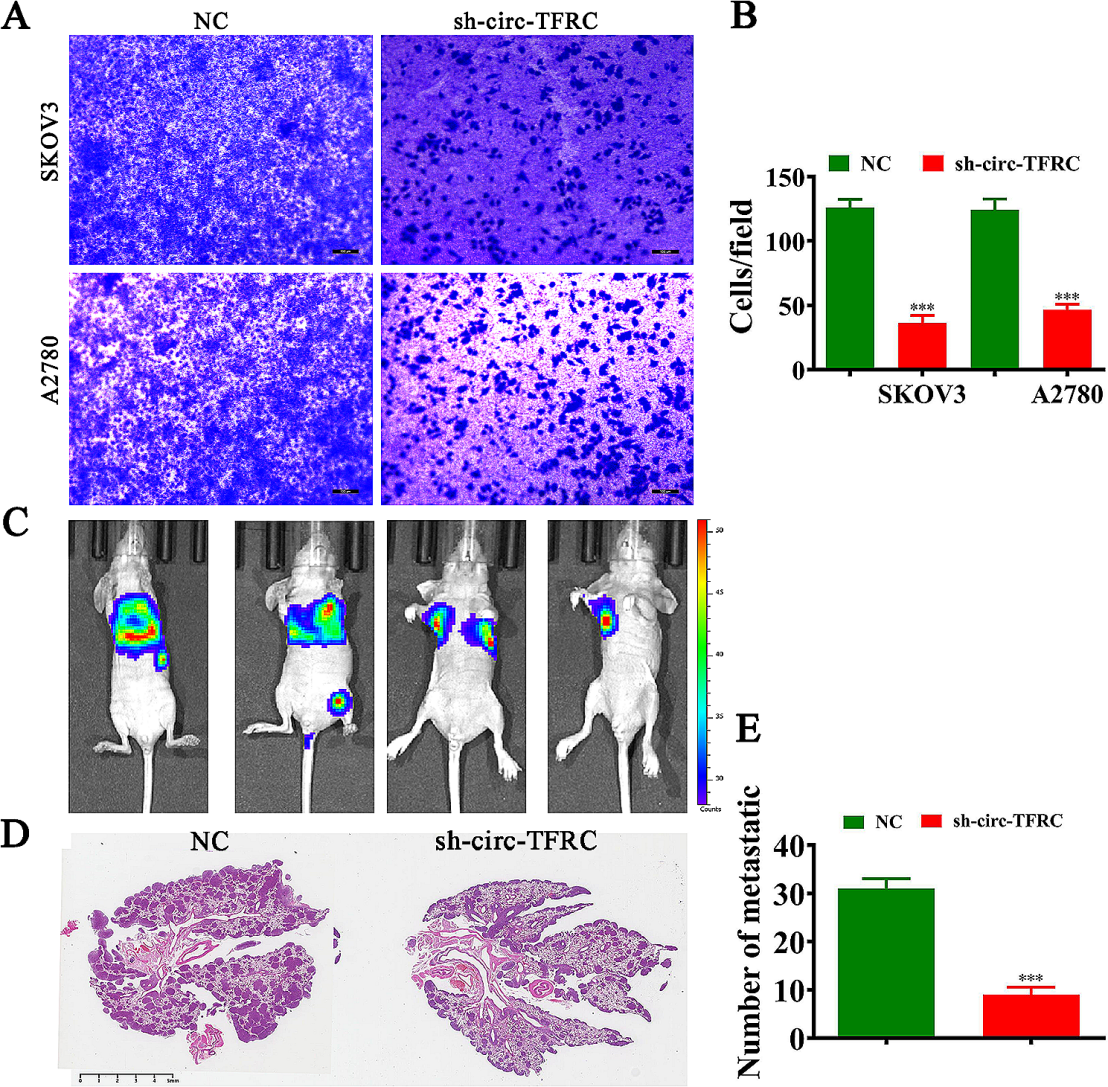
Downregulation of circ-TFRC inhibited OC cell migration and tumor lung metastasis. (**A** and **B**) Transwell assay showed the migration of both SKOV3 and A2780 cells. The data are expressed as the mean ± SD. ^***^P < 0.001 versus NC. *n* = 3. (**C**) Live imaging detection showed the pulmonary metastasis of SKOV3 cells. *n* = 5. (**D** and **E**) Numbers of metastatic foci in lung tissues were calculated based on the HE staining. The data are expressed as the mean ± SD. ^***^P < 0.001 versus NC. *n* = 3

Transwell migration assay and invasion detections showed that circ-TFRC downregulation suppressed A2780 and SKOV3 cell invasions and migrations (Fig. 3A, B). Living image detection demonstrated pulmonary metastasis of SKOV3 cells, revealing that circ-TFRC silencing decremented pulmonary metastasis capability via reducing the metastatic foci numbers in lung tissues post HE staining (Fig. 3C and E). This advised that circ-TFRC downregulation inhibited OC invasion.

### Mir-615-3p and IGF2 were circ-TFRC downstream targets

**Fig. 4:**
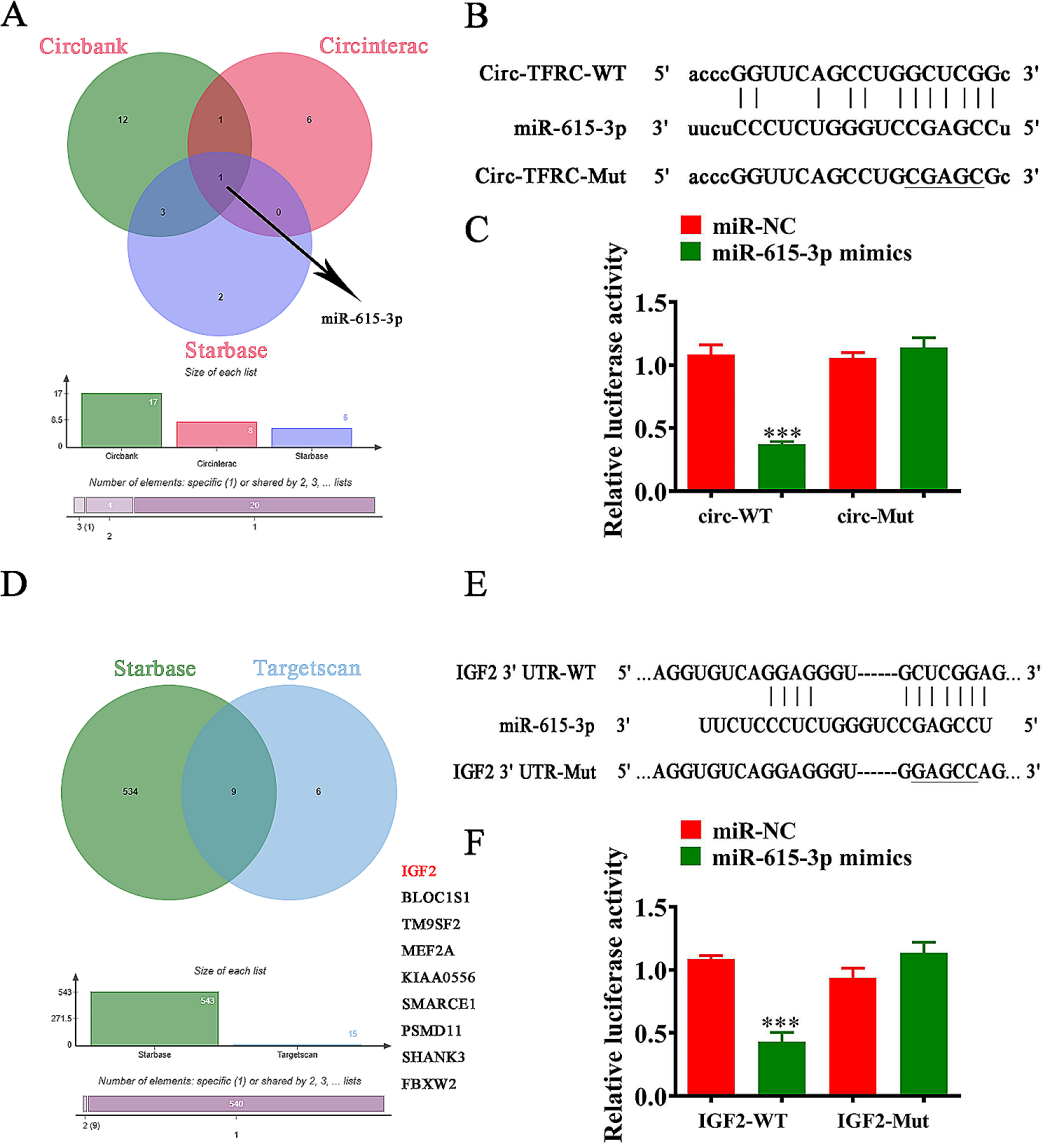
miR-615-3p and IGF2 were the downstream targets of circ-TFRC. (**A**) Bioinformatics analysis showed the downstream target of circ-TFRC using different websites. (**B** and **C**) Luciferase activity of circ-TFRC in HEK293T cells transfected with miR-615-3p mimics, which were putative binding sites for the circ-TFRC sequence. Luciferase activity was normalized by Renilla luciferase activity. *n* = 3. (**D**) Bioinformatics analysis showed the downstream target of miR-615-3p using different websites. (**E** and **F**) Relative luciferase activity determined 48 h after the transfection of HEK293T cells with miR-615-3p mimic/NC or IGF2-3’UTR WT/Mut. The data are expressed as mean ± SD. ^***^P < 0.001. *n* = 3

The analyses found that circ-TFRC can interacted with different miRNAs, but only miR-615-3p could interact with circ-TFRC (Fig. 4A). We prepared luciferase reporter vector that transfected with various miRNA mimics. Outcomes validated that miR-615-3p could reduce fluorescein intensity significantly, implying that miR-615-3p was circ-TFRC downstream target (Fig. 4B, C).

The bioinformatics data revealed that BLOC1S1, IGF2, TM9SF2, MEF2A, KIAA0556, SMARCE1, PSMD11, SHANK3, and FBXW2 were miR-615-3p downstream targets, with only IGF2 having more conservative combination with miR-615-3p. We converted Wt or mut 3’UTR-IGF2 sequences such like miR-615-3p-binding sequence into luciferase reporter vector to further verify correlations between miR-615-3p and IGF2 (Fig. 4D). Technician transfected luciferase reporter vector into HEK293 cells with or not miR-23b-3p mimic. Data revealed that miR-615-3p inhibited luciferase activity in Wt yet not mut cells (Fig. 4E).

### IGF2 overexpression or mir-615-3p inhibition reversed OC cell migration after circ-TFRC silencing

**Fig. 5:**
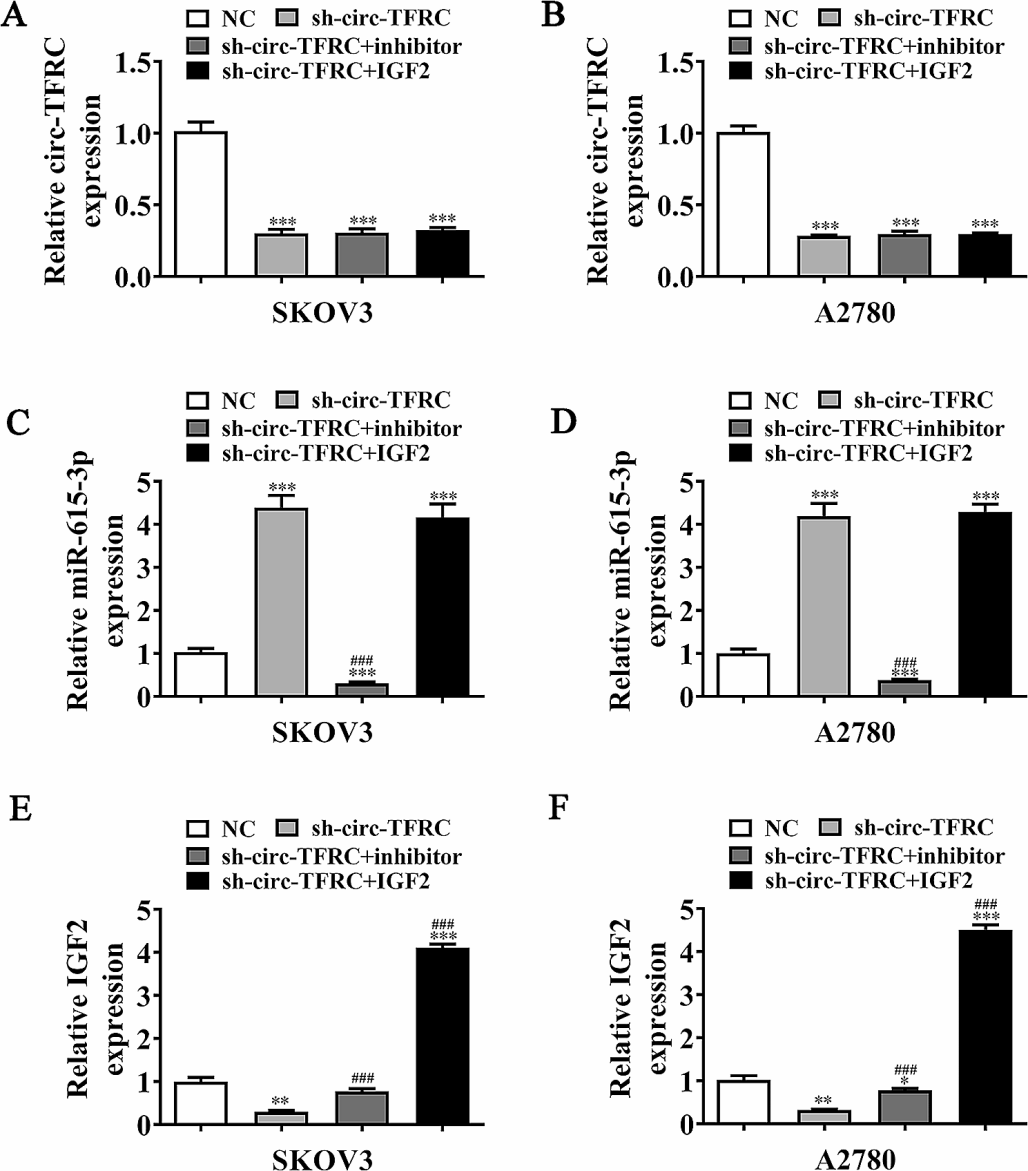
Intermolecular regulation among circ-TFRC, miR-615-3p, and IGF2. (**A**–**F**) RT-qPCR detection assay showed the expression of circ-TFRC, miR-615-3p, and IGF2 in both SKOV3 and A2780 cells transfected with sh-circ-TFRC, circ-TFRC inhibitor, and IGF2-overexpressing single or multiple vectors. The data are expressed as mean ± SD. ^*^P < 0.05, ^**^P < 0.01, ^***^P < 0.001 versus NC. ^###^*P* < 0.001 versus sh-circ-TFRC. *n* = 3

RT-qPCR outcomes indicated that circ-TFRC expression decremented after circ-TFRC-silencing vector transfections. miR-615-3p suppressor or IGF2 overexpression treatments did not affect circ-TFRC expression in A2780 and SKOV3 cells (Fig. 5A, B), suggesting that miR-615-3p and IGF2 were at circ-TFRC downstream. Rt-qPCR outputs revealed that circ-TFRC silencing incremented miR-615-3p expression. IGF2 overexpression had no effects upon sh-circ-TFRC-induced miR-615-3p expression (Fig. 5C, D), implying that miR-615-3p was at the circ-TFRC downstream. The results showed that circ-TFRC silencing decreased IGF2 expression. Nevertheless, suppressing miR-615-3p reversed sh-circ-TFRC effects upon IGF2 expression. IGF2 expression increased significantly after IGF2-overexpressing vector transfections (Fig. 5E and F), implying that circ-TFRC promoted IGF2 expression through miR-615-3p sponging.

**Fig. 6:**
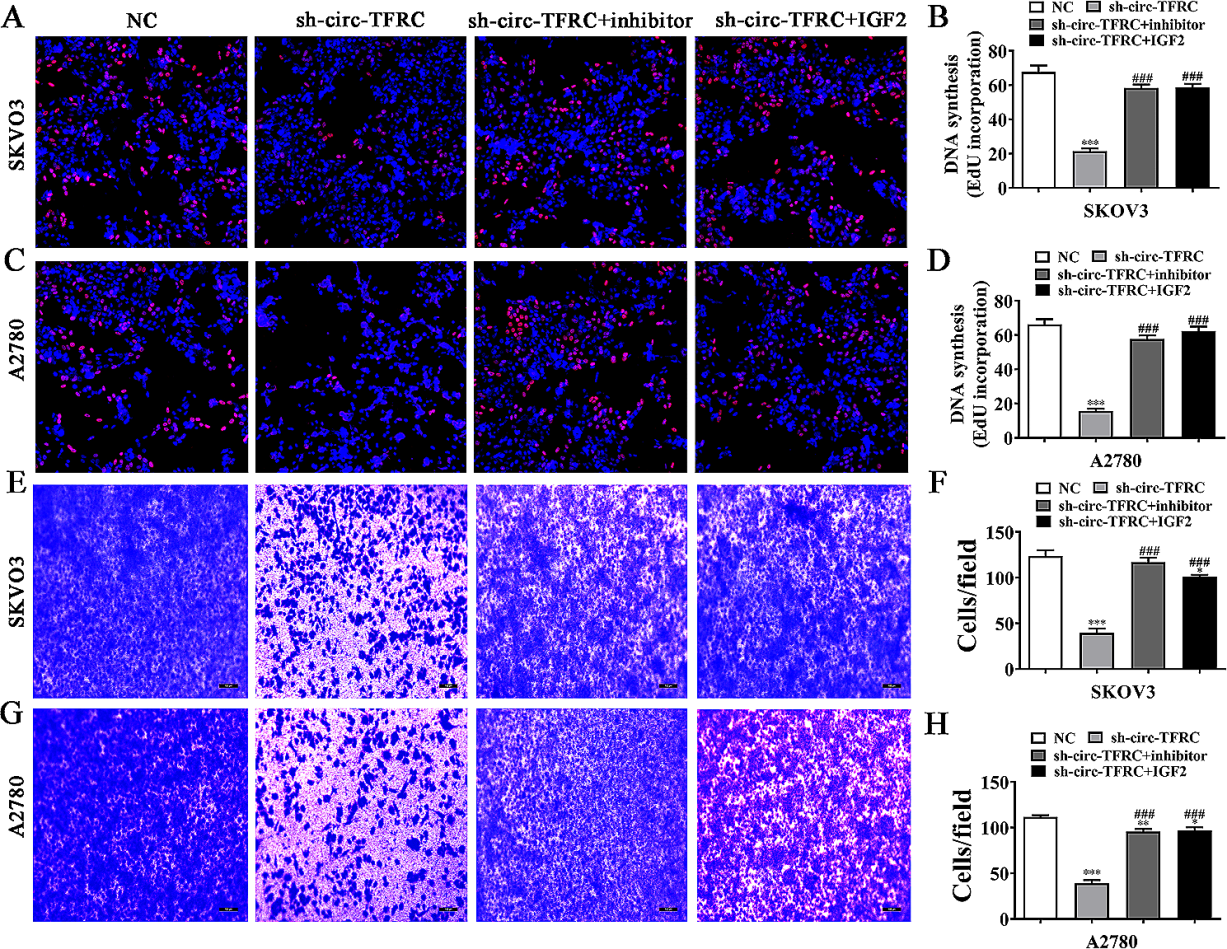
Overexpression of IGF2 or inhibition of miR-615-3p reversed OC cell proliferation and migration after circ-TFRC silencing. (**A**–**C**) EdU detection showed the proliferation ability of both SKOV3 and A2780 cells. The data are expressed as the mean ± SD. ^***^P < 0.001 versus NC. ^###^*P* < 0.001 versus sh-circ-TFRC. *n* = 3. (**D**–**F**) Transwell assay showed the invasion and migration of SKOV3 and A2780 cells. The data are expressed as the mean ± SD. ^*^P < 0.05, ^**^P < 0.01, ^***^P < 0.001 versus NC. ^##^*P* < 0.01, ^###^*P* < 0.001 versus sh-circ-TFRC. *n* = 3

EdU detection illustrated that IGF2 overexpression or miR-615-3p suppression restored OC cell proliferation ability after circ-TFRC silencing (Fig. 6A and D). Transwell migration assay showcased that IGF2 overexpression or miR-615-3p suppression restored OC cell migration in A2780 and SKOV3 cells post circ-TFRC silencing (Fig. 6E and H).

### IGF2 overexpression reversed OC cell proliferation post mir-615-3p upregulation

**Fig. 7:**
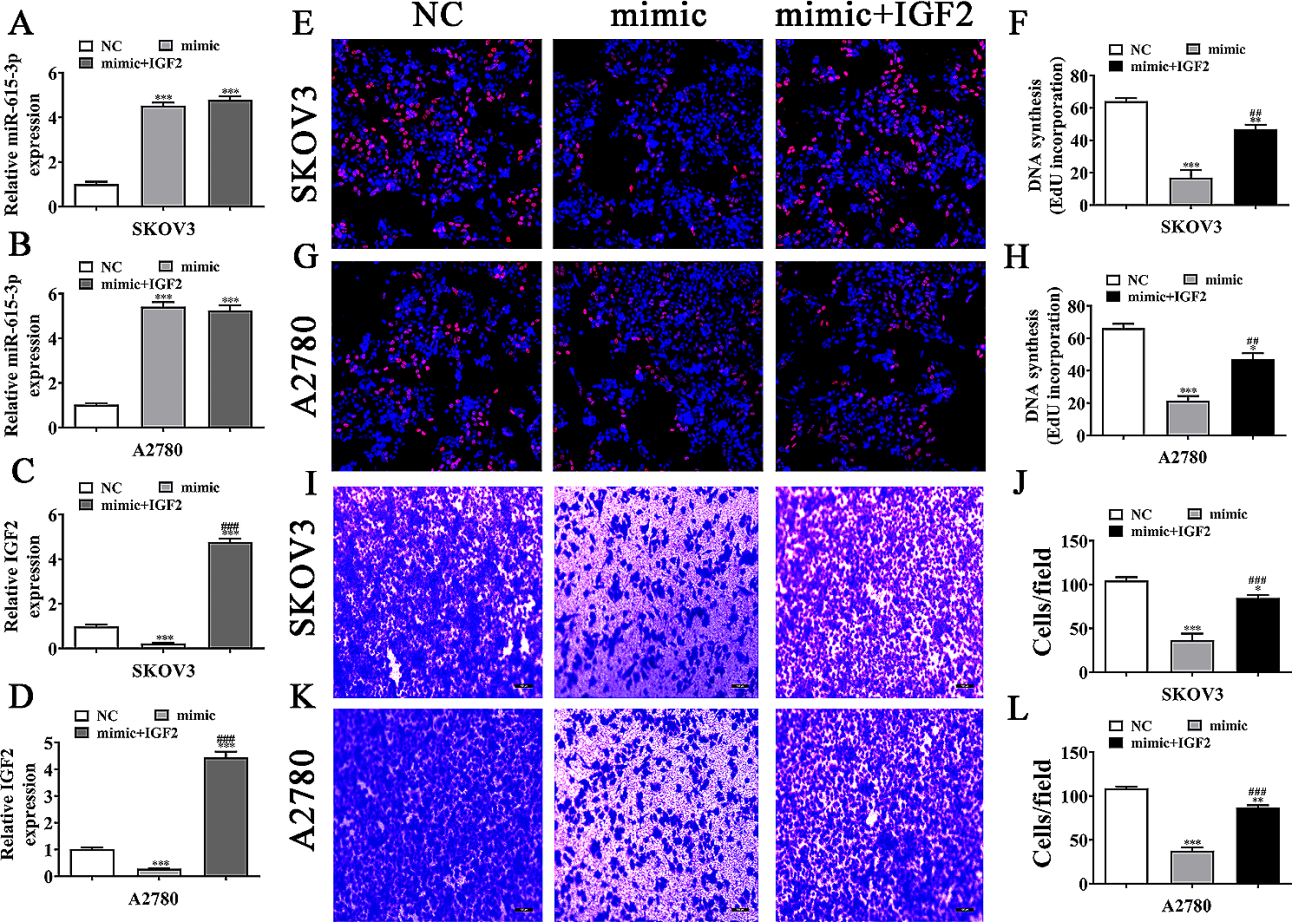
Overexpression of IGF2 reversed OC cell proliferation and migration after miR-615-3p upregulation. (**A**–**D**) RT-qPCR detection showed the expression of miR-615-3p and IGF2 in both SKOV3 and A2780 cells. The data are expressed as the mean ± SD. ^***^P < 0.001 versus NC. ^###^*P* < 0.001 versus mimic. *n* = 3. (**E**–**H**) EdU detection showed the proliferation ability of both SKOV3 and A2780 cells. The data are expressed as the mean ± SD. ^*^P < 0.05, ^**^P < 0.01, ^***^P < 0.001 versus NC. ^##^*P* < 0.01 versus mimic. *n* = 3. (I–L) Transwell assay showed the invasion and migration of SKOV3 and A2780 cells. The data are expressed as the mean ± SD. ^*^P < 0.05, ^**^P < 0.01, ^***^P < 0.001 versus NC. ^###^*P* < 0.001 versus mimic. *n* = 3

We also revealed that miR-615-3p expression was increased after transfected with miR-615-3p mimic in both A2780 and SKOV3 cells (Fig. 7A and B). IGF2 upregulation could not reverse the miR-615-3p expression. However, miR-615-3p overexpression inhibited IGF2 expression. IGF2 expression increased significantly in A2780 and SKOV3 cells after transfecting with IGF2-overexpressing heterologous vector (Fig. 7C and D).

EdU detection showed that IGF2 overexpression restored OC cell proliferations post miR-615-3p upregulation (Fig. 7E and H). Transwell migration assay illustrated that IGF2 overexpression restored OC cell migration post miR-615-3p upregulation (Fig. 7I and L).

## Discussion

Former research confirmed that circRNA has significant role during OC progression [17, 18]. Our study found that circ-TFRC expression enhanced in OC cells and tissues, suggesting that high circ-TFRC expression indicated a poor prognosis of OC. Further, we found in experiments that circ-TFRC downregulation inhibited cell migration, validating circ-TFRC functions during OC progression.

A number of studies revealed that circRNA could regulate gene expression by miRNA sponging [19]. The present study employed bioinformatics analysis to examine whether circ-TFRC acted with miR-615-3p, which we validated through luciferase report analysis. Former studies found that high miR-615-3p expression reduced migration, invasion, and proliferation abilities in different cancers, such as cervical and lung cancers [20–22]. This study also verified miR-615-3p roles as OC tumor inhibitor. miR-615-3p overexpression suppressed NSCLC cell growth [23]. This study found that circ-TFRC silencing promoted miR-615-3p expression. Inhibiting miR-615-3p restored cell migrations post circ-TFRC silencing.

Other research revealed that miR-615-3p could interact with IGF2 3’ UTR. IGF2 is a 7.5-kDa mitogenic peptide hormone expressed in many tissues. IGF2 gene expression is strictly regulated. Its overexpression in various tumors is correlated with poor prognosis [24–26]. This study revealed that circ-TFRC silencing downregulated IGF2 expression. The IGF2 overexpression reimposed cell proliferation and migration post circ-TFRC silencing. Furthermore investigations unraveled that miR-615-3p overexpression inhibited IGF2 expression. The IGF2overexpression reimposed cell migration and proliferation post miR-615-3p upregulation.

Overall, this study revealed that circ-TFRC increased IGF2 expression by sponging miR-615-3p to exert tumorigenic effects. Thus, we identified circ-TFRC/miR-615-3p/IGF2 loop and its mechanisms and biological effects in malignant OC. This loop could function as a candidate therapeutic marker of OC.

## Data Availability

The datasets used and/or analyzed during the current study are available from the corresponding author on reasonable request.
